# System of Diseases of the Eye

**Published:** 1898-09

**Authors:** 


### Book Reviews.

---

SYSTEM OF DISEASES OF THE EYE. By American, British, Dutch, French, German and Spanish authors. Edited by WILLIAM F. NORRIS, A. M., M. D., and CHARLES A. OLIVER, A. M., M. D., of Philadelphia. Vol. III. Local Diseases, Glaucoma, Wounds and Injuries, Operations, with 50 full page plates and 186 text illustrations. Octavo. Pages XII.—962. Philadelphia: J. B. Lippincott & Co. 1898.

The third volume of this elaborate work deals with subjects which pertain more directly to clinical ophthalmology than those of the preceding volumes. It embodies local diseases, glaucoma, wounds and injuries, operations, the local diseases, however, not including those of the cornea, crystalline lens and muscles. The latter, among other subjects, are reserved for the fourth and concluding volumes. This volume is considerably larger than the preceding ones and is printed, illustrated and put together in the same excellent style. There are numerous full-page plates, many of which are colored, together with a large number of cuts, all of which are beautifully executed and add greatly to the value of the text.

That the volume is a most important addition to ophthalmological literature is guaranteed by the names of its contributors, including as they do some of the most eminent writers and practitioners in Europe and America. There is some overlapping of subjects, but this is not at all objectionable in such a work as this, as the views of different men of wide experience on a given subject are often desirable. The proportionate length, detail and character of the articles vary also, some being theoretical, others practical, some historical and bibliographical, others limited to the personal views and practice of the writers. But this, too, is to be expected. Notwithstanding this lack of uniformity and symmetry of the articles, each is excellent in its way and each is authoritative and trustworthy, but some are better than others, according to the standpoint from which they are viewed by the reader.

The first article of the volume is by Charles Stedman Bull, of New York, on Diseases of the orbit, and occupies sixty pages. It includes anomalies of the orbit, lesions of its bony walls, injuries, orbital cellulitis, inflammation of the capsule of Tenon, thrombosis of the orbital veins and cavernous sinus, enophthalmus, pulsating exophthalmos, tumors and diseases of the cavities adjacent to and secondarily involving the orbit. The subjects are treated comprehensibly and in that concise and clear style characteristic of Dr. Bull.

The second and third articles, of 26 and 44 pages respectively, are on Diseases of the eyelids and operations performed upon the eyelids, by George C. Harlan, of Philadelphia, Pa. Dr. Harlan, also clear and concise in his statements, gives an excellent résumé of the diseases which are met with in the eyelids and of the various operations that may be performed upon them. The articles are in some respects rather brief, but this is in keeping with the importance of the subject.

Diseases of the lachrymal apparatus, 40 pages, by Samuel Theobold, of Baltimore, Md., first reviews the anatomy of the lachrymal gland and passages and then describes their diseases. Dr. Theobold has given much study to this subject and handles it familiarly. In overcoming strictures of the nasal ducts, he uses his well-known large probes, and emphasises their superior value by a large and successful experience with them.

The next article, of 82 pages, is on Diseases of the conjunctiva and sclera, by Swan M. Burnett, of Washington, D. C., and is exceedingly practical and valuable. The ground, as regards etiology, pathology and treatment, is well covered and the latest conclusions are introduced. The section on ophthalmia neonatorum is especially important and should be read, not only by ophthalmologists, but by obstetricians as well. We believe that this disease and its frightful consequences could be prevented, almost without exception, if every obstetrician would adopt the practice here recommended.

W. A. Brailey and Sydney Stephenson, of London, Eng., have contributed an excellent article of 80 pages on Diseases of the iris and ciliary body. The descriptions are full and clear and most important topics are discussed. The subjects are considered under the following general heads: The clinical examination of the iris and the ciliary body, inflammation of the iris and the ciliary body, degenerations of the iris, tumors and parasites of the iris and the ciliary body, and disorders of movement of the iris. Were there space, it would be interesting to note the leading points and conclusions of the authors, but it must suffice to refer to but one. Keratitis punctata is regarded not as a keratitis or iritis, but as a cyclitis, the little dots on the posterior surface of the cornea being deposits which are thrown, as it were, against this surface from the aqueous humor, and which come in the first place from an inflamed ciliary body. This view is undoubtedly correct, but it is at variance with that heretofore held.

The next contribution is by A. Hill Griffith, of Manchester, Eng. It covers 60 pages and deals with Diseases of the choroid and vitreus in a most judicial manner. Some new topics are introduced, and all are discussed in the light of the latest discoveries.

In the space of 18 pages, Isador Schnabel, of Vienna, elaborates a new theory of the anatomy of staphyloma posticum and the relationship of the condition to myopia. He defends most ably the proposition that every posterior conus (staphyloma) is congenital, a malformation and not a consequence of disease; the conus, so small at birth as to be scarcely distinguishable from a ring of normal connective tissue, increasing in size with the eye. The antero-posterior axis, and therefore the refraction, or myopia, increase with it.

Diseases of the retina is a long chapter of 166 pages by Joseph Schöbl, of Prague. All the different forms of retinal diseases are here described in great detail.

Following this article is a comprehensive, though less detailed, discussion of Diseases of the optic nerve, extending over 50 pages, by Johann Deyl, of Prague. It is divided into sections which treat respectively of circulatory and functional disturbances, affecting the papilla of the optic nerve, inflammation of the optic nerve, atrophy of the papilla of the optic nerve and congenital anomalies and tumors of the optic nerve. The two articles may be regarded as a treatise in themselves, worthy of most careful study.

Glaucoma: Pathogenesis, symptoms, course and treatment, is the topic of the next section of the book, 56 pages, and is succinctly described by Priestly Smith, of Birmingham, Eng., who has made it a life-long study. His well-known views on the etiology of glaucoma are set forth, and the treatment of the disease is in accordance with the most experienced practitioners of the present time.

Next we come to Wounds and injuries of the eyeball and its appendages, a subject of extreme practical interest to every practitioner. Emil Gruening, of New York, is one of our most experienced and discriminating American ophthalmologists and he writes as one having authority. In thirty-six pages he gives an excellent résumé of the injuries of the eye, classifying them into those by contusion, by penetration, by penetration with retention of foreign body, and by light, heat and chemical substances. His descriptions are good and the treatment recommended is sound. The part devoted to injuries by penetration with retention of the foreign body is especially to be commended.

Robert Randolph, of Baltimore, gives a very complete review of Sympathetic ophthalmia in 56 pages, dealing with it historically as well as systematically under the heads of definition, etiology, varieties, symptoms, diagnosis, course, pathogenesis, prognosis and treatment. He devotes considerable attention to the question of pathogenesis and fails to find evidence to corroborate Deutschmann's migratory theory and is unable even to verify his experiments. The various methods of preventing and treating the disease are fully described. The concluding article of the book is somewhat lengthy, occupying as it does 164 pages, and embodies a concise and practical description of operations usually performed in eye surgery by that most skilful and experienced ophthalmic surgeon, Herman Knapp, New York, whose fame as an ophthalmologist is world-wide. All the operations which the author considers most proper in eye-surgery are taken up, the indications for them given and the methods of performing them described, together with the preparations and precautions which should be taken and the results and complications which may follow. Dr. Knapp has managed to compress into this article a vast amount of practical information, and the whole of it is backed by a personal experience which but few men in this department of surgery have acquired. Not every one who studies the methods of operating preferred and executed by Dr. Knapp will adopt them, but all will concede to him an authority which could be accorded to but few in this or any other country. None should fail to learn what this master of our art has to say in this crystallisation of his vast experience and indefatigable study.

Taking the volume as a whole, it is a splendid presentation of the more practical side of ophthalmology, and embodies the latest accepted views and conclusions in pathology, etiology and treatment. Too much cannot be said in its praise. A. A. H.

